# Late neurodegeneration in the Tau transgenic mouse model rTg4510

**DOI:** 10.1186/1750-1326-8-S1-P58

**Published:** 2013-10-04

**Authors:** Lone Helboe, Christiane Volbracht

**Affiliations:** 1 Neuroscience Drug Discovery, H. Lundbeck, Valby, Denmark

## Background

The rTg4510 mouse overexpresses an inducible human mutant tau (TauP301L) selectively in the forebrain. This mouse exhibits Tau hyperphosphorylation at AD-relevant epitopes, accumulation of the 64 kDa tau species, and neurofibrillary tangles in cortex and hippocampus.

## Materials and methods

The rTg4510 mice express the TauP301L mutation under control of a tetracycline-responsive operon promoter. rTg4510, single transgenic tTA and non-transgenic littermates (129S6/FVB/N F1 hybrid) were bred at Taconic, Denmark. Paraffin sections were stained for p-Tau, astro- and microglial markers, ubiquitin, doublecortin and tangles. Western blot was performed on soluble (S1) and sarkosyl-insoluble (P3) brain fractions. Hippocampal CA1 neuron numbers and cortical neuronal densities were assessed by un-biased stereology at Gubra, Denmark.

## Results

rTg4510 mice exhibited age-dependent progression of hyperphosphorylated Tau as well as gliosis and ubiquitination. Phosphorylated Tau (p-Tau) was first detected in cortical neurites (from 10 weeks of age) and then followed by p-Tau in cell bodies as well as in the hippocampus. The 64 kDa Tau species were present from 16W. By stereology, we detected a significant decrease in CA1 neuron number compared to both non-transgenic and tTA mice from 10W. The CA1 neuron number remained constant in rTg4510 until 32W of age. By 48W, the neuronal number was decreased by 40%, thus pointing to a late onset of neurodegeneration in our cohort of rTg4510 mice. The neuronal density in cortex was increased at 32W compared to 10W suggesting a loss of neurites and accompanying synapses. To further investigate a potential developmental effect in our transgenic mice, brain sections from all 3 genotypes were stained for doublecortin. We observed an aberrant staining pattern of newborn neurons in the subgranular zone in rTg4510 as well as tTA compared to non-transgenics.

## Conclusions

Our cohort of rTg4510 mice displays a pattern of tau pathology that is comparable to what has previously been reported in this transgenic mouse strain (1). Further, gliosis and ubiquitination coincide with appearance of hyperphosphorylated Tau. This phenotype is reversed by suppression of Tau transgene expression with doxycycline. By stereological assessment of CA1 neuron number, we detect no progression in cell loss in rTg4510 mice until between 32 and 48 weeks of age. However, at 10 weeks rTg4510 neuron number is already differentiated from control mice. Thus, we suggest that our cohort of rTg4510 mice exhibit late neurodegeneration induced by pathological Tau species whereas early cell loss may be inflicted by developmental changes due to the overexpression of Tau transgene.
